# Supplementary material for: Unveiling the Bioactive Potential of the Invasive Jellyfish Phyllorhiza punctata Through Integrative Transcriptomic and Proteomic Analyses
Source: Biomolecules. 2025 Aug 4;15(8):1121. doi: 10.3390/biom15081121 (PMC12383608; doi:10.3390/biom15081121)
Supplement: Supplementary file 1 [file biomolecules-15-01121-s001.zip › Suplementary Material/FileS3/PEPFOLD4/XP_065069170_5763/clusters.html]

 PEP-FOLD clusters


Download archive of all models  here   

|  |  |  |
| --- | --- | --- |
| # | file | sOPEP |
| # cluster 1 - Size: 49 |  |  |
|  | XP\_065069170\_5763-model1 | -24.553 |
|  | XP\_065069170\_5763-model1.1 | -24.4291 |
|  | XP\_065069170\_5763-model1.2 | -24.4066 |
|  | XP\_065069170\_5763-model1.3 | -24.3592 |
|  | XP\_065069170\_5763-model1.4 | -24.2481 |
|  | XP\_065069170\_5763-model1.5 | -24.2388 |
|  | XP\_065069170\_5763-model1.6 | -24.2187 |
|  | XP\_065069170\_5763-model1.7 | -24.2046 |
|  | XP\_065069170\_5763-model1.8 | -24.1845 |
|  | XP\_065069170\_5763-model1.9 | -24.1772 |
|  | XP\_065069170\_5763-model1.10 | -24.1764 |
|  | XP\_065069170\_5763-model1.11 | -24.165 |
|  | XP\_065069170\_5763-model1.12 | -24.1431 |
|  | XP\_065069170\_5763-model1.13 | -24.1113 |
|  | XP\_065069170\_5763-model1.14 | -24.0959 |
|  | XP\_065069170\_5763-model1.15 | -24.093 |
|  | XP\_065069170\_5763-model1.16 | -24.0914 |
|  | XP\_065069170\_5763-model1.17 | -24.0854 |
|  | XP\_065069170\_5763-model1.18 | -24.0768 |
|  | XP\_065069170\_5763-model1.19 | -24.0668 |
|  | XP\_065069170\_5763-model1.20 | -24.0312 |
|  | XP\_065069170\_5763-model1.21 | -24.018 |
|  | XP\_065069170\_5763-model1.22 | -24.009 |
|  | XP\_065069170\_5763-model1.23 | -23.9957 |
|  | XP\_065069170\_5763-model1.24 | -23.995 |
|  | XP\_065069170\_5763-model1.25 | -23.9925 |
|  | XP\_065069170\_5763-model1.26 | -23.9808 |
|  | XP\_065069170\_5763-model1.27 | -23.97 |
|  | XP\_065069170\_5763-model1.28 | -23.9648 |
|  | XP\_065069170\_5763-model1.29 | -23.9593 |
|  | XP\_065069170\_5763-model1.30 | -23.9569 |
|  | XP\_065069170\_5763-model1.31 | -23.9506 |
|  | XP\_065069170\_5763-model1.32 | -23.878 |
|  | XP\_065069170\_5763-model1.33 | -23.8735 |
|  | XP\_065069170\_5763-model1.34 | -23.8578 |
|  | XP\_065069170\_5763-model1.35 | -23.8497 |
|  | XP\_065069170\_5763-model1.36 | -23.8424 |
|  | XP\_065069170\_5763-model1.37 | -23.8418 |
|  | XP\_065069170\_5763-model1.38 | -23.7936 |
|  | XP\_065069170\_5763-model1.39 | -23.788 |
|  | XP\_065069170\_5763-model1.40 | -23.7569 |
|  | XP\_065069170\_5763-model1.41 | -23.7465 |
|  | XP\_065069170\_5763-model1.42 | -23.7421 |
|  | XP\_065069170\_5763-model1.43 | -23.7332 |
|  | XP\_065069170\_5763-model1.44 | -23.7274 |
|  | XP\_065069170\_5763-model1.45 | -23.718 |
|  | XP\_065069170\_5763-model1.46 | -23.7071 |
|  | XP\_065069170\_5763-model1.47 | -23.6782 |
|  | XP\_065069170\_5763-model1.48 | -23.6054 |
| # cluster 2 - Size: 13 |  |  |
|  | XP\_065069170\_5763-model2 | -18.4385 |
|  | XP\_065069170\_5763-model2.1 | -18.356 |
|  | XP\_065069170\_5763-model2.2 | -18.3117 |
|  | XP\_065069170\_5763-model2.3 | -18.3013 |
|  | XP\_065069170\_5763-model2.4 | -18.2639 |
|  | XP\_065069170\_5763-model2.5 | -18.2539 |
|  | XP\_065069170\_5763-model2.6 | -18.0332 |
|  | XP\_065069170\_5763-model2.7 | -18.007 |
|  | XP\_065069170\_5763-model2.8 | -17.7826 |
|  | XP\_065069170\_5763-model2.9 | -17.6914 |
|  | XP\_065069170\_5763-model2.10 | -17.5724 |
|  | XP\_065069170\_5763-model2.11 | -17.5383 |
|  | XP\_065069170\_5763-model2.12 | -17.3991 |
| # cluster 3 - Size: 7 |  |  |
|  | XP\_065069170\_5763-model3 | -17.8686 |
|  | XP\_065069170\_5763-model3.1 | -17.8603 |
|  | XP\_065069170\_5763-model3.2 | -17.8008 |
|  | XP\_065069170\_5763-model3.3 | -17.3793 |
|  | XP\_065069170\_5763-model3.4 | -17.3268 |
|  | XP\_065069170\_5763-model3.5 | -17.0716 |
|  | XP\_065069170\_5763-model3.6 | -16.9667 |
| # cluster 4 - Size: 5 |  |  |
|  | XP\_065069170\_5763-model4 | -17.757 |
|  | XP\_065069170\_5763-model4.1 | -17.7218 |
|  | XP\_065069170\_5763-model4.2 | -17.7154 |
|  | XP\_065069170\_5763-model4.3 | -17.5885 |
|  | XP\_065069170\_5763-model4.4 | -17.2675 |
| # cluster 5 - Size: 12 |  |  |
|  | XP\_065069170\_5763-model5 | -17.6438 |
|  | XP\_065069170\_5763-model5.1 | -17.1893 |
|  | XP\_065069170\_5763-model5.2 | -16.8106 |
|  | XP\_065069170\_5763-model5.3 | -16.7269 |
|  | XP\_065069170\_5763-model5.4 | -16.6923 |
|  | XP\_065069170\_5763-model5.5 | -16.6749 |
|  | XP\_065069170\_5763-model5.6 | -16.6525 |
|  | XP\_065069170\_5763-model5.7 | -16.6057 |
|  | XP\_065069170\_5763-model5.8 | -16.5925 |
|  | XP\_065069170\_5763-model5.9 | -16.5536 |
|  | XP\_065069170\_5763-model5.10 | -16.4729 |
|  | XP\_065069170\_5763-model5.11 | -16.4694 |
| # cluster 6 - Size: 12 |  |  |
|  | XP\_065069170\_5763-model6 | -17.4212 |
|  | XP\_065069170\_5763-model6.1 | -17.0554 |
|  | XP\_065069170\_5763-model6.2 | -17.0519 |
|  | XP\_065069170\_5763-model6.3 | -17.0312 |
|  | XP\_065069170\_5763-model6.4 | -16.9096 |
|  | XP\_065069170\_5763-model6.5 | -16.7913 |
|  | XP\_065069170\_5763-model6.6 | -16.7373 |
|  | XP\_065069170\_5763-model6.7 | -16.7162 |
|  | XP\_065069170\_5763-model6.8 | -16.6824 |
|  | XP\_065069170\_5763-model6.9 | -16.6012 |
|  | XP\_065069170\_5763-model6.10 | -16.5176 |
|  | XP\_065069170\_5763-model6.11 | -16.5078 |
| # cluster 7 - Size: 1 |  |  |
|  | XP\_065069170\_5763-model7 | -16.5156 |
| # cluster 8 - Size: 1 |  |  |
|  | XP\_065069170\_5763-model8 | -16.2954 |
|  |  |  |
